# Nacre-Inspired Composite Coatings with Hierarchical Architecture for Durable Surface Protection

**DOI:** 10.1021/acs.chemmater.5c02825

**Published:** 2026-02-13

**Authors:** Aranzazu Sierra-Fernández, Diego Cortes, Miguel A. Monclus, Kenneth J.T. Livi, Michael Kappl, Stefan A.L. Weber, D. Howard Fairbrother, Rafael Fort

**Affiliations:** † Institute of Geosciences (CSIC, UCM), C/ Severo Ochoa 7, Madrid 28040, Spain; ‡ Department of Chemistry, 1466 Johns Hopkins University, 3400 N Charles Street, Baltimore, Maryland 21218, United States; § 9149 Max Planck Institute for Polymer Research, Ackermannweg 10, Mainz 55128, Germany; ∥ 202528 IMDEA Materials Institute, C/Eric Kandel 2, Getafe, Madrid 28906, Spain; ⊥ Department of Earth and Planetary Sciences, Johns Hopkins University, Baltimore, Maryland 21218, United States; # Materials Characterization and Processing, Johns Hopkins University, 800 Wyman Park Dr., Baltimore, Maryland 21211, United States; ∇ Department of Physics, University of Mainz, Staudingerweg 7, Mainz 55128, Germany; ○ Institute for Photovoltaics, University of Stuttgart, Pfaffenwaldring 47, Stuttgart 70569, Germany

## Abstract

A bioinspired multilayer coating is developed for the protection of built cultural heritage, emulating the hierarchical architecture of natural nacre. The system is fabricated through the alternating deposition of mineralized calcium carbonate (CaCO_3_) and organic layers composed of chitosan and cellulose nanofibrils (CNFs), with poly­(acrylic acid) (PAA) acting as a mineralization-directing agent. A CO_2_-controlled environment promotes the formation of continuous crystalline CaCO_3_ layers with strong interfacial adhesion to marble substrates. The resulting composite multilayers exhibit stratified organization and mechanical properties comparable to those of the biogenic minerals. Nanoindentation and stiffness mapping reveal hardness and modulus values in the range of natural nacre, along with enhanced reinforcement with increasing numbers of multilayers. Mechanical durability under acidic conditions confirms the preservation of both structural integrity and aesthetic compatibility, with color changes remaining below perceptual thresholds (ΔEab < 5). The observed crack resistance, cohesive strength, and mechanical compatibility with the substrate highlight the effectiveness of the layered architecture for dissipating stress and inhibiting damage propagation. These results contribute to the development of an emerging class of bioinspired protective coatings that integrate mechanical resilience, chemical stability, and visual compatibility by establishing a groundwork for advanced materials tailored to the complex demands of cultural heritage conservation.

## Graphical abstract

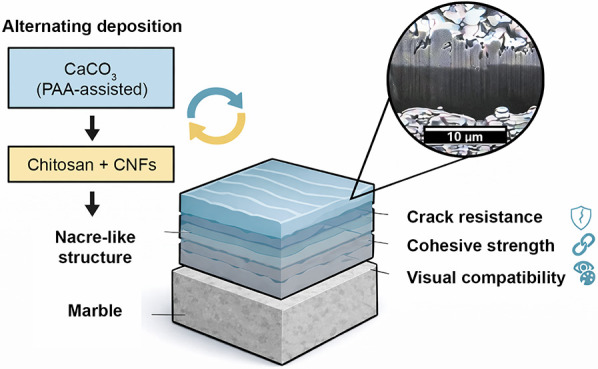

## 1 Introduction

Built cultural heritage is naturally vulnerable to degradation owing to its continuous exposure to environmental stressors. Key factors contributing to this deterioration include water infiltration, which induces physical damage, predominantly through salt crystallization processes. These processes generate crystallization pressures within the pore structure, leading to internal stress development and subsequent structural degradation;^1,2^ temperature fluctuations, which induce expansion and contraction cycles;^3^ and biological colonization by fungi, algae, and lichens, which contribute to both biochemical alteration of the substrate and mechanical damage through hyphal penetration and biofilm development.^4,5^ These degradation phenomena are increasingly intensified by climate change, as extreme rainfall events enhance water ingress and salt transport, whereas prolonged droughts exacerbate drying cycles and crystallization stresses, ultimately accelerating material loss.^6^ In parallel, climate-driven ground subsidence and sea-level rise further threaten the structural stability of historic buildings, particularly in coastal areas.^7,8^ Consequently, the conservation of historic stone architecture has become an urgent scientific and societal challenge.

To mitigate these risks, a wide range of protective treatments have been developed, including silanes,^9,10^ polymeric coatings such as epoxies^11^ and polyurethanes,^12^ ceramics,^13,14^ and more recently, nanotechnology-based materials.^15^ Despite decades of research and application, these approaches generally provide only partial and often short-term solutions. Synthetic polymeric resins, including acrylics, vinyl polymers, organosilicon compounds, and fluorinated materials, have been extensively employed as consolidants and protective agents for stone substrates.^15,16^ However, their limited physicochemical compatibility with mineral surfaces frequently results in undesirable effects, such as surface whitening, cracking, pore blocking, or reduced vapor permeability, ultimately compromising durability and aesthetic integrity.^17^ Nanostructured coatings incorporating metal oxide nanoparticles or polymer nanocomposites have demonstrated improved functionality;^15,18,19^ however, their long-term stability, UV resistance, scalability, and potential environmental or health impacts remain insufficiently understood.^20–22^ Inorganic treatments, such as hydroxyapatite, offer improved chemical affinity with carbonate stones and enhanced resistance to acidic environments.^23,24^ However, as with many mineral-based approaches, their overall performance depends on the processing conditions and environmental exposure, which may influence their long-term effectiveness in certain applications.^25^ Overall, despite significant progress, existing protective systems remain limited in their ability to simultaneously fulfill the mechanical, chemical, aesthetic, and sustainability requirements of architectural heritage conservation.

A fundamental limitation underlying many of these approaches is the difficulty of designing coatings that combine high stiffness (i.e., the ability of the material to resist deformation) with effective energy dissipation (related to the material’s toughness), properties that are traditionally mutually exclusive.^26^ For stone conservation, coatings must resist mechanical stresses without cracking or delaminating, while remaining compatible with the substrate and preserving its visual appearance. Addressing this mechanical trade-off requires strategies that integrate both material composition and structural design.

Nature offers compelling solutions to these challenges. Nacre, the inner layer of mollusk shells, exemplifies how the hierarchical integration of stiff mineral components with a compliant organic matrix can result in exceptional mechanical performance. Composed of approximately 95 vol % calcium carbonate (CaCO_3_) and 5 vol % organic biopolymers,^27,28^ nacre achieves an exceptional balance between strength and toughness.^29,30^ Its brick-and-mortar architecture, consisting of highly oriented aragonite tablets embedded within organic layers, enables efficient load transfer, energy dissipation, and crack deflection.^30–33^ This synergy between composition and architecture provides a powerful design paradigm for overcoming the classical mechanical trade-offs in synthetic materials.

Recent advances in bioinspired materials have demonstrated that these principles can be translated into artificial systems, including nacre- and enamel-inspired composites that combine rigid mineral frameworks with deformable intergranular phases.^34–36^ These materials exhibit remarkable mechanical properties, validating the potential of hierarchical design, interfacial engineering, and multiscale organization in their development. However, most of these systems are developed as freestanding materials, and their translation into thin, conformal, and substrate-compatible coatings remains largely unexplored.

In particular, replicating nacre-like architectures directly onto stone substrates poses significant challenges, as natural nacre undergoes complex biomineralization pathways that are difficult to reproduce under controlled conditions.^30,37,38^ Achieving continuous, oriented mineral layers within an organic matrix requires precise control over mineral growth, polymer organization, and interfacial interactions.^39–41^

Herein, we report the fabrication of a bioinspired multilayer coating that emulates the hierarchical structure of nacre and is directly mineralized on marble substrates. By combining mineralized CaCO_3_ layers with organic nanostructures through a controlled layer-by-layer process, this approach contributes to addressing the stiffness-toughness trade-off while ensuring physicochemical compatibility and mechanical robustness in mineral-based materials.

## 2 Experimental Section

### 2.1 Materials

Chitosan (medium molecular weight, high degree of deacetylation ≥75%; Sigma-Aldrich), ammonium bicarbonate (NH_4_HCO_3_, ≥99.0%; Sigma-Aldrich), poly­(acrylic acid) (PAA; *M*_w_ = 1800 g/mol; Sigma-Aldrich), poly­(l-glutamic acid) (PGlu; *M*_w_ = 13,000 Da; Alamanda Polymers), CaCl_2_ (≥99%; Sigma-Aldrich), and NaOH (≥97%; Sigma-Aldrich) were used as received. A 3.0 wt % aqueous gel of cellulose nanofibrils (CNF) was obtained from the Product Development Center (PDC) at the University of Maine (Orono, Maine, USA). Ultrapure Millipore water was used for all the experiments. Nonpolished test cubes, each measuring 1 cm^3^, were prepared from raw quarry slabs of Olympian White Danby marble. The marble was sourced from a quarry in Vermont, USA, known for supplying much of the marble used in American architectural and sculptural heritage.

### 2.2 Fabrication of the Polymer-Coated Surfaces

Chitosan was dissolved at a concentration of 2% w/v in 1% v/v acetic acid to use as the interfacial layer to initiate CaCO_3_ mineralization. Thin chitosan films were deposited via spin coating. Specifically, 30 μL of chitosan–acetic acid solution was deposited onto a clean 1 × 1 cm marble substrate, followed by spin coating at 5000 rpm for 1 min. Subsequently, the chitosan-coated marble substrates were submerged in a 4% w/v NaOH solution for 10 min to neutralize the protonated amino groups and prevent further dissolution.^42^ In this immersion step, the stone samples were covered with Teflon to ensure that the coating was deposited on only one side of the sample. Following this step, the coated substrates were thoroughly rinsed with deionized water to remove any residual NaOH, and then dried at ∼37 °C. The resulting coated substrates were used directly in subsequent experiments without additional treatment.

### 2.3 Biomineralization on Polymer-Coated Surfaces

In a typical procedure, the calcitic layer on the chitosan-coated samples was growth through a controlled mineralization process using CO_2_ generated from the decomposition of ammonium bicarbonate (NH_4_HCO_3_) via the ammonium diffusion method. Prior to mineralization, the chitosan-coated substrates were covered with Teflon, exposing only one face. A precursor solution was then prepared by using a CaCO_3_ precursor solution consisting of 20 mM calcium chloride (CaCl_2_) and 40 μg/mL poly­(acrylic acid) (PAA) as a low-dose mineralization modifier. The chitosan-coated substrates were then submerged face-up in 10 mL of this precursor solution in 25 mL vials. Each vial was covered with aluminum foil, and punctured with a single perforation to allow controlled gas diffusion. The vials containing the immersed samples were placed on a ceramic plate in the upper compartment of a desiccator. Approximately 2 g of NH_4_HCO_3_ was placed at the bottom of the desiccator to generate CO_2_. The desiccator was sealed and incubated at 25 ± 1 °C. Each mineralization step was carried out over 24 h, allowing the CO_2_ to diffuse and promote the mineral growth on the exposed face of the chitosan-coated substrates. To construct multilayered systems, a 1.0 wt % CNF suspension was used to create intermediate layers between successive chitosan coatings. Both chitosan and CNF layers were deposited using the same conditions: 30 μL of solution was applied onto a clean 1 × 1 cm marble substrate, followed by spin coating at 5000 rpm for 1 min. After each layer was deposited, the samples were dried under ambient conditions before proceeding to the next layer. This alternating deposition of chitosan and CNF was repeated to build multilayered organic scaffolds. The final mineralization step was then carried out on the outermost chitosan layer following the procedure described above.

### 2.4 Other Related Experiments

To investigate the significance of the mineralization solution precursor and the role of chitosan as an interfacial layer for mineralization, experiments were conducted using different concentrations of PAA, specifically 40 μg/mL, and 200 μg/mL. A comparative study was also performed in the absence of chitosan and polymer additives. To explore the importance of polymer additives on the morphological differences of the crystalline CaCO_3_ formed, a set of experiments was conducted using poly­(l-glutamic acid) at a concentration of 1 g L^–1^.

### 2.5 Structural, Chemical and Mineralogical Characterization

The X-ray diffraction (XRD) patterns of the samples were recorded using an X-ray diffraction (XRD, Bruker D8-Advance, Cu Kα = 1.54 Å, Germany) in the Grazing Incidence XRD geometry. The X-ray source was operated at a voltage of 40 keV and current of 40 mA. The scans were performed over a range of 10 to 70° using a scintillator detector with a scan rate of 5°/min and a step size of 0.01°, and an incident angle α of 0.5. Fourier transform infrared (FTIR) spectra of the samples were obtained by Fourier transform infrared spectroscopy with attenuated total reflectance accessories (ATR-FTIR, Nicolet iS5 spectrometer and an iD5 ATR attachment, Thermo Fisher Scientific, USA). Raman spectra were obtained using a laser confocal Raman spectrometer (BWS475-785-S, Raman i-Pro, BWTEK, USA) equipped with a 532 nm excitation laser. Spectra were acquired in the range of 100–1800 cm^–1^, using a laser power of 5 mW at the sample surface to avoid thermal damage. Each spectrum was obtained by averaging 3 scans with an integration time of 10 s per scan. Calibration was performed using a standard silicon wafer peak at 520 cm^–1^. All measurements were performed at room temperature under ambient conditions. The morphologies and structures of the samples were determined by scanning electron microscopy (JSM IT100, JEOL, Japan). A 3D Laser Scanning Microscope (KEYENCE VK-X200) was used to measure the surface roughness of the samples. The analysis of samples for cross section were prepared using a focused ion beam (FIB) and analyzed with a FIB-SEM system (Helios G5 UC Focused Ion Dual Beam, Thermo Fisher Scientific, USA). Optical microscope images of crossed polarizers were obtained using a polarizing microscope (Zeiss Axiophot, Germany) equipped with a Leica color CCD camera to analyze the textural features of thin-sections. For this petrographic analysis polished thin sections measuring ca. 25 μm thick were prepared. Atomic force microscopy (AFM) images of the samples were collected by using an Oxford Instruments/Asylum Research MFP-3D Infinity AFM, within a nitrogen glovebox (humidity level below 0.3% and oxygen level below 0.1%) for all experiments.

### 2.6 Mechanical Characterization

To evaluate the mechanical performance of the composite multilayer coatings (CML), two complementary techniques were used: nanoindentation, which probes hardness (i.e., resistance to permanent deformation) and elastic modulus (i.e., resistance to elastic deformation), and peak force quantitative nanomechanical (PF-QNM) mapping. PF-QNM allows simultaneous acquisition of topographical and mechanical maps, specifically, local stiffness expressed as Young’s modulus, within the same area. This facilitates the visualization of local spatial variations in mechanical response arising from the microstructural heterogeneity of the layered system.

#### 2.6.1 Nanoindentation

Nanoindentation was carried out by using a Triboindenter TI-950 nanoindenter (Hysitron-Bruker) equipped with a diamond Berkovich indenter. The tests were conducted in a load-controlled mode using a load function comprising a 10 s load, 10 s hold, and 5 s unload, with a maximum load of 30 mN. Multiple indentations were performed at different locations with a minimum separation distance of 20 μm. The reported values for nanoindentation hardness (*H*) and reduced modulus (*E^r^*) represented the average of at least 10 indents, and were obtained using the Oliver and Pharr method^43^ by analyzing the load–displacement curves generated during the tests. To obtain the elastic modulus, the unloading portion of the load-depth curve was analyzed according to

$$E^{r}=\frac{S\sqrt{\pi }}{2\sqrt{A\left(h\right)}}$$

where *A*(*h*) is the projected area of contact, obtained from the tip area function, which was calibrated beforehand from indentations on a fused silica standard sample of known modulus, *S* is the contact stiffness, and *E* ^*r*^ is the reduced elastic modulus. The hardness (*H*) was determined from the peak load (*F*_max_) and *A*(*h*) as

$$H=\frac{F_{\max}}{A\left(h\right)}$$

#### 2.6.2 Scratch Tests

The same Triboindenter TI-950 system was used to perform scratch tests across the substrate/CML interface using constant loads of 75 and 100 mN. Scratches were performed using a 10 μm radius spherical diamond tip for 30 s up to a final length of 40 μm.

#### 2.6.3 Peak Force Quantitative Nanomechanical Mapping (PF-QNM)

PF-QNM was performed using a Dimension FastScan AFM (Bruker, Santa Barbara, CA, USA) equipped with a diamond tip cantilever (model D300 probes, ART). The resonance frequency of the cantilever was 325 kHz, with a spring constant of 40 N/m and a nominal radius of 5–10 nm. AFM imaging was conducted in air at a scan rate of 0.1–0.3 Hz, and the loading forces during measurements were maintained between 1000 and 2000 nN. The diamond tip cantilever was calibrated initially on sapphire surfaces to set the deflection sensitivity. Once calibrated, the tip was used for PF-QNM imaging. Force curves and images were obtained using the Peakforce Capture function to probe microscale and nanoscale morphology and mechanical properties. The Derjaguin–Muller–Toporov (DMT) model was used to calculate the average elastic moduli from the force–displacement curves obtained by PF-QNM. This model is suitable for describing tip–sample interactions dominated by elastic contact with long-range adhesive forces, and is commonly applied in AFM-based mechanical mapping of stiff materials with low surface adhesion. Assuming that the measured values followed a normal distribution, we fitted a Gaussian function to the values recorded for each material. Additionally, height images were acquired to evaluate the surface morphology.

#### 2.6.4 Sample Preparation

Samples for nanoindentation and PF-QNM were embedded in a two-component epoxy resin (Araldit 2020), sectioned using a water-cooled diamond saw. The samples were ground to expose the marble-coating interface, and polished with 1 and 0.3 μm aluminum oxide abrasives. The final thin sections were mounted on glass slides for analysis. Additionally, samples were prepared both perpendicular and parallel to the layered structure for mechanical characterization, allowing for the measurement of mechanical properties both in the cross-section and along the surface. For reference, a fragment of *Haliotis ovina* (abalone) nacre was prepared following the same protocol and included as a natural example of a well-characterized hierarchical composite. Although the microstructure of natural nacre is not identical to that of the synthetic coatings, both systems share the same fundamental architectural principle, consisting of stiff mineral lamellae separated by thin organic interfaces. This brick-and-mortar architecture underlies nacre’s exceptional mechanical performance and provides a mechanically relevant benchmark for the composite multilayer coatings (CMLs), which alternate mineralized CaCO_3_ layers with polymer-rich interlayers. Accordingly, this nacre system serves as an appropriate reference for contextualizing the mechanical performance of synthetic layered composites, in line with established practice in biomimetic and hybrid-material studies.^44^

### 2.7 Acid-Resistance Experiments

To evaluate acid resistance, duplicate coated samples were exposed to controlled acid conditions. The tests were conducted at pH 5 and 25 °C under continuous stirring to ensure uniform exposure. Prior to each experiment, the pH meter was calibrated using standard buffer solutions at pH 4.0, 7.0, and 10.0. The pH of the test solution was adjusted to 5.0 using nitric acid (HNO_3_).

### 2.8 Color Changes

Color measurements were performed by diffuse reflectance using a spectrophotometer (MINOLTA CM-700d, Tokyo, Japan) across the visible spectrum (400–700 nm) in accordance with ASTM E313-00 (2000), using 10 nm intervals and a spectral resolution of 0.01%. Measurements were conducted under D65 illumination with a 10° standard observer angle and a 3 mm aperture mask. Three replicates were performed for each treated and untreated sample. The instrument calibration was performed using a white reference cap (CM-A177). The color coordinates recorded included L* (lightness, 0 = black, 100 = white), a* (red-green axis), and b* (yellow-blue axis).

The total color difference (ΔE*ab) between the treated and untreated surfaces was calculated as follows:

$$\Delta E^{*}ab=\sqrt{\left(\Delta L^{2}+\Delta a^{2}+\Delta b^{2}\right)}$$

Additionally, the chroma (C*ab), which quantifies color saturation, was determined as follows:

$$C^{*}=\sqrt{a^{*2}b^{*2}}$$

This parameter reflects the intensity of perceived color and complements ΔE*ab when assessing visual impact.

## 3 Results and Discussion

### 3.1 Fabrication of Individual Mineral Composite Layers

In this study, poly­(acrylic acid) (PAA) was selected as the primary additive because of its high affinity for calcium ions and ability to act as a templating agent, promoting the nucleation and growth of well-defined mineral phases.^45–47^ In addition to its effectiveness in regulating CaCO_3_ mineralization, PAA is a water-soluble polyelectrolyte commonly employed in polymer-assisted crystallization studies.^47^ In the present system, PAA is used in small amounts and is expected to be largely immobilized within the CaCO_3_ layer, thereby limiting potential environmental release. Biobased mineralization modifiers, such as alginate, carboxymethyl cellulose, or polypeptides including poly­(l-glutamic acid), also provide effective Ca^2+^-binding functionalities and represent promising alternatives for further increasing the renewable content of similar systems.^47,48^ The mineralization process was performed on marble substrates previously coated with a chitosan layer deposited under mild conditions to ensure homogeneous surface coverage. Using a controlled CO_2_ diffusion method, the mineralization process on a marble substrate was triggered, leading to the progressive formation of a chitosan–CaCO_3_ composite layer, as shown in Figure 1. The mineralization process is initiated with the electrostatic adsorption of the negatively charged PAA onto the positively charged chitosan surface. Chitosan, a cationic biopolymer containing hydroxyl (−OH) and primary amine (−NH_2_) groups, became protonated (NH_3_^+^) under acidic conditions, enhancing its interaction with PAA.^49^ The next step involves the addition of Ca^2+^ ions. During this biomineralization process, the carboxyl (−COOH) groups of PAA, anchored to the chitosan layer deposited on the marble surface, act as nucleation sites for Ca^2+^ ion coordination (Figure 1a). The sequential adsorption of PAA and Ca^2+^ was performed once to create a uniform PAA–Ca^2+^ complex layer. This initial layer underwent mineralization under a controlled CO_2_ atmosphere, which triggered the transformation of the amorphous precursor into a crystalline CaCO_3_ film (Figure 1b). The resulting chitosan–CaCO_3_ composite exhibited densely packed crystalline domains with structural homogeneity resembling that of naturally occurring biominerals.^50,51^ As shown in the scanning electron microscopy (SEM) image (Figure 1b), the mineralized overlayer exhibited a cohesive and continuous morphology, indicative of a well-developed mineralization process.

**Figure 1:**
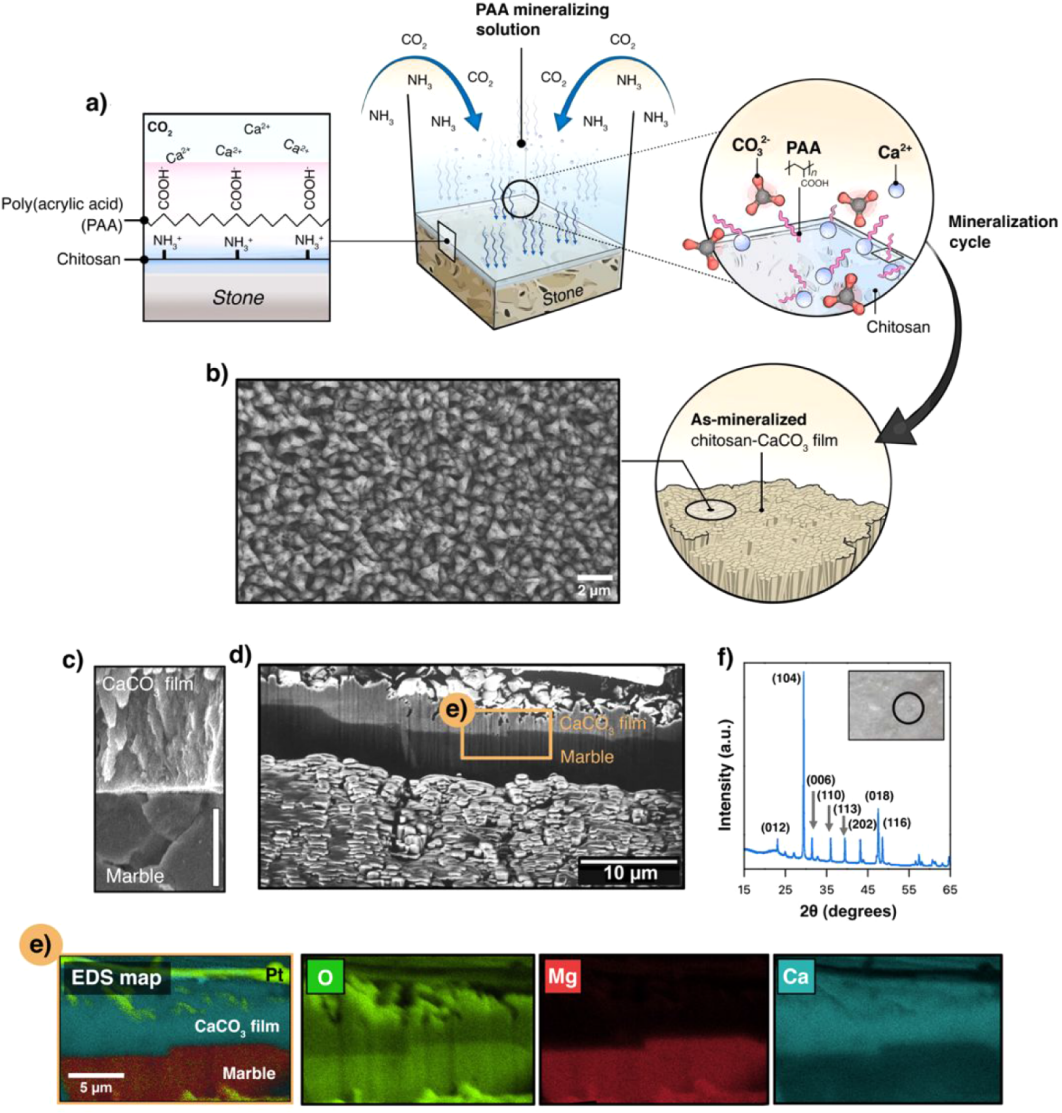
Schematic and characterization of the chitosan–CaCO_3_ mineralized layer on marble substrate. (a) Illustration of the mineralization process using a PAA mineralizing solution, highlighting the adsorption of poly­(acrylic acid) (PAA) onto the chitosan surface and the subsequent interaction with Ca^2+^ ions under a controlled CO_2_ environment. (b) SEM image of the chitosan–CaCO_3_ film surface showing densely packed crystalline domains. (c) Cross-sectional SEM image of the CaCO_3_ film and dolomitic marble interface. Scale bar: 3 μm. (d) Cross-sectional FIB-SEM view of the mineralized layer over marble substrate. (e) EDS elemental mapping of the cross-section showing the distribution of oxygen (O), magnesium (Mg), and calcium (Ca) within the composite. (f) XRD pattern of the CaCO_3_ film confirming the presence of calcite, with a notable reflection along the (104) plane. The inset shows the analyzed area.

Previous studies^52^ have emphasized that achieving a continuous and smooth chitosan layer is essential to promote uniform mineral nucleation and growth, leading to the formation of structurally homogeneous organic–inorganic hybrid coatings.^52^ In line with this, our scanning electron microscopy (SEM) observations revealed that the use of a nonuniform chitosan/PAA seed layer, characterized by the presence of pores and discontinuities, led to poorly organized and irregular mineralized structures (Figure S1, Supporting Information). The occurrence of such defective seed layer was primarily associated with slight deviations in deposition parameters, such as chitosan concentration or applied volume. Importantly, this variability was readily mitigated by optimizing the coating conditions, which enabled the reproducible formation of homogeneous, continuous interfacial layers suitable for controlled mineralization. Polymer additives also play a pivotal role in shaping the structural characteristics of mineralized layers.^53,32^ To further explore this, poly-l-glutamic acid (PGlu), a polypeptide rich in carboxylic acid groups, was tested as an alternative additive to PAA because of its reported effectiveness in influencing mineralization outcomes.^48^ As shown in the Supporting Information, the use of 1 g L^–1^ PGlu as an additive resulted in the formation of spherulitic microdomains composed of granular constituents over the marble substrate (Figure S2, Supporting Information). This morphology, characterized by a rougher and more irregular surface texture, suggests that PGlu induces a particle-involved mineralization pathway, producing a crystalline product with a mesoscopic texture which differed significantly from the more controlled structures obtained with PAA over marble substrates (Figure 1b).

Further experiments were conducted to examine the effects of mineralization in the absence of any polymer additives as well as with varying concentrations of PAA (Figure S3, Supporting Information). The strong dependence of CaCO_3_ morphology on PAA concentration (Figure S3) can be rationalized in terms of the influence of PAA on Ca^2+^ availability, nucleation density, and crystal growth kinetics during mineralization. At low PAA concentrations (40 μg mL^–1^), partial Ca^2+^ complexation and surface-associated polymer chains likely promote heterogeneous nucleation and stabilize transient amorphous calcium carbonate precursors, resulting in finely structured and more uniformly distributed crystallites that coalesce into a continuous mineralized layer. Using this same PAA concentration, a qualitatively similar mineralization behavior was observed in a single experiment performed on calcite substrates (Figure S4, Supporting Information), suggesting that the mineralization mechanism identified here is not strictly limited to marble substrates, although systematic substrate-dependent studies are required to fully generalize this behavior. In contrast, at higher PAA concentrations (200 μg mL^–1^) (Figure S3c, Supporting Information), increased Ca^2+^ complexation in solution may reduce the effective supersaturation near the substrate and limit the secondary nucleation events. Under these conditions, crystal growth appears to be dominated by fewer nuclei, leading to the formation of larger and more angular CaCO_3_ crystals and discontinuous mineral domains. In the absence of polymer additives, mineralization proceeds via comparatively unregulated crystallization, yielding sparsely distributed rhombohedral calcite crystals. These observations are consistent with polymer-mediated nonclassical crystallization pathways, in which the balance between ion complexation, precursor stabilization, and surface-confined nucleation critically determines mineral morphology.^32,47,53,54^

Further structural insights are provided by the cross-sectional SEM image in Figure 1c, which reveals the clear delineation between the newly formed CaCO_3_ film and the underlying marble substrate. This stratification highlights the successful deposition of the mineral layer, adhering firmly to the substrate. In Figure 1d, a broader FIB cross-sectional view shows the layer thickness of 3.52 ± 0.65 μm across different batches, indicating the good reproducibility of the CO_2_ diffusion-driven mineralization process. The relatively narrow thickness distribution reflects the controlled nature of both CO_2_ generation and PAA-mediated nucleation, which were kept constant in terms of precursor concentration, exposure time, temperature and reactor geometry. In addition to thickness reproducibility, SEM observations revealed a laterally continuous and homogeneous mineralized layer across the substrate surface for all batches prepared under identical conditions, indicating that the CO_2_ diffusion-driven and PAA-mediated process yields consistent coverage and uniformity. To assess the elemental composition and distribution within the composite structure, energy-dispersive X-ray spectroscopy (EDS) mapping was performed on the FIB cross section of the sample (Figure 1e). The analysis revealed that calcium was predominantly localized within the biomineralized surface layer, indicating its association with the CaCO_3_ phase formed during the mineralization process. In contrast, magnesium was primarily concentrated in the underlying dolomitic marble substrate (Figure 1e and Figure S5, Supporting Information) consistent with the determination that all of the marble cubes used in this study were predominantly dolomitic, as evidenced by the XRD pattern and EDS point analyses shown in Figure S5. Minor calcitic inclusions were observed, but they did not dominate the overall mineralogical composition. Oxygen was homogeneously distributed across both the mineralized layer and substrate (Figure 1e), consistent with the presence of carbonate groups (CO_3_^2–^) in both the CaCO_3_ film and dolomitic marble. The elemental mapping results, therefore, show distinct compositional layers within the composite, with the Ca-rich layer corresponding to mineralized CaCO_3_ and the Mg-rich region representing the underlying dolomitic marble substrate. This structural arrangement is further corroborated by X-ray diffraction (XRD) analysis, which confirms that the CaCO_3_ phase corresponds to calcite, with a notable reflection along the (104) plane, as shown in Figure 1f. Moreover, Raman spectroscopy (Figure S6, Supporting Information) revealed distinct vibrational modes characteristic of the calcite phase, with the symmetric stretching mode of the carbonate ions at 1088 cm^–1^ exhibiting the highest intensity. Additional peaks observed at 153 cm^–1^, 282 cm^–1^, and 712 cm^–1^ further confirm the purity of the calcite structure, with no evidence of secondary phases.

### 3.2 Composite Multilayer Coating (CML) Approach

In this study, cellulose nanofibrils (CNF) were used as an interfacial component between the mineralized-CS layers (Figure 2a) motivated by their reported high mechanical strength, flexibility, and the ability to form a dense nanofibrillar network.^55,56^ The morphological evolution of the surface throughout the coating process is shown in Figure 2b–e. Initially, the surface of the mineralized-CS layer (Figure 2b) displayed a granular texture, characteristic of calcium carbonate deposition, which was further confirmed by FTIR spectrum (Figure 2b, *bottom*) showing prominent carbonate (CO_3_^2–^) vibrational bands, including the v_3_, v_2_, and v_4_ vibrational bands of CO_3_^2–^. The v_3_ band, associated with the asymmetric stretching of CO_3_^2–^, appeared around 1400–1500 cm^–1^, while the v_2_ and v_4_ modes, related to out-of-plane and in-plane bending, respectively, were observed at 870 cm^–1^ and 700 cm^–1^. These findings support the data discussed in the previous paragraph that indicate the presence of calcite in the mineralized layer. Following the introduction of the CNF layer, the SEM images revealed noticeable smoothing of the surface, attributed to the deposition of fibrous cellulose conforming to the underlying mineralized topography (Figure 2c). While some larger surface features remained visible, their softened outlines suggested uniform CNF coverage across the mineral surface. The introduction of CNFs was supported by the FTIR spectra, which showed additional bands corresponding to O–H stretching (3000–3500 cm^–1^) and C–O vibrations (1063 cm^–1^) associated with cellulose (Figure 2c, *bottom*). Elemental analysis further indicated an increase in the carbon content relative to that of the initial mineralized CS–CaCO_3_ layer (Figure 2c, *inset*). The CNF layer was deposited under the same controlled spin-coating conditions used for chitosan (30 μL, 5000 rpm, 1 min), ensuring consistent application throughout the multilayer system. The subsequent deposition of the CS layer (Figure 2d) further refined the surface appearance by enhancing the continuity and coverage of the organic matrix. SEM analysis revealed a more homogeneous topography, and elemental analysis showed an increase in both carbon and oxygen signals, consistent with the incorporation of the organic chitosan matrix (Figure 2d, *inset*). FTIR identified characteristic amide bands (1650 cm^–1^ and 1550 cm^–1^), confirming the presence of the chitosan matrix, along with a reduction in the relative intensity of the v_4_ CO_3_^2–^ band (700–725 cm^–1^), suggesting partial masking of the underlying mineralized CS–CaCO_3_ surface (Figure 2d, *bottom*). Finally, the mineralization of the CS layer (Figure 2e) restored the granular texture observed in the initial mineralization stage (Figure 2b). This was corroborated by FTIR spectra, which showed an increase in the relative intensity of the carbonated bands associated with calcite (Figure 2e, *bottom*), indicating renewed mineral growth on the topmost layer. The enhanced carbonate signal, relative to the underlying organic bands, supports successful mineralization beyond the initial CS–CaCO_3_ layer.

**Figure 2:**
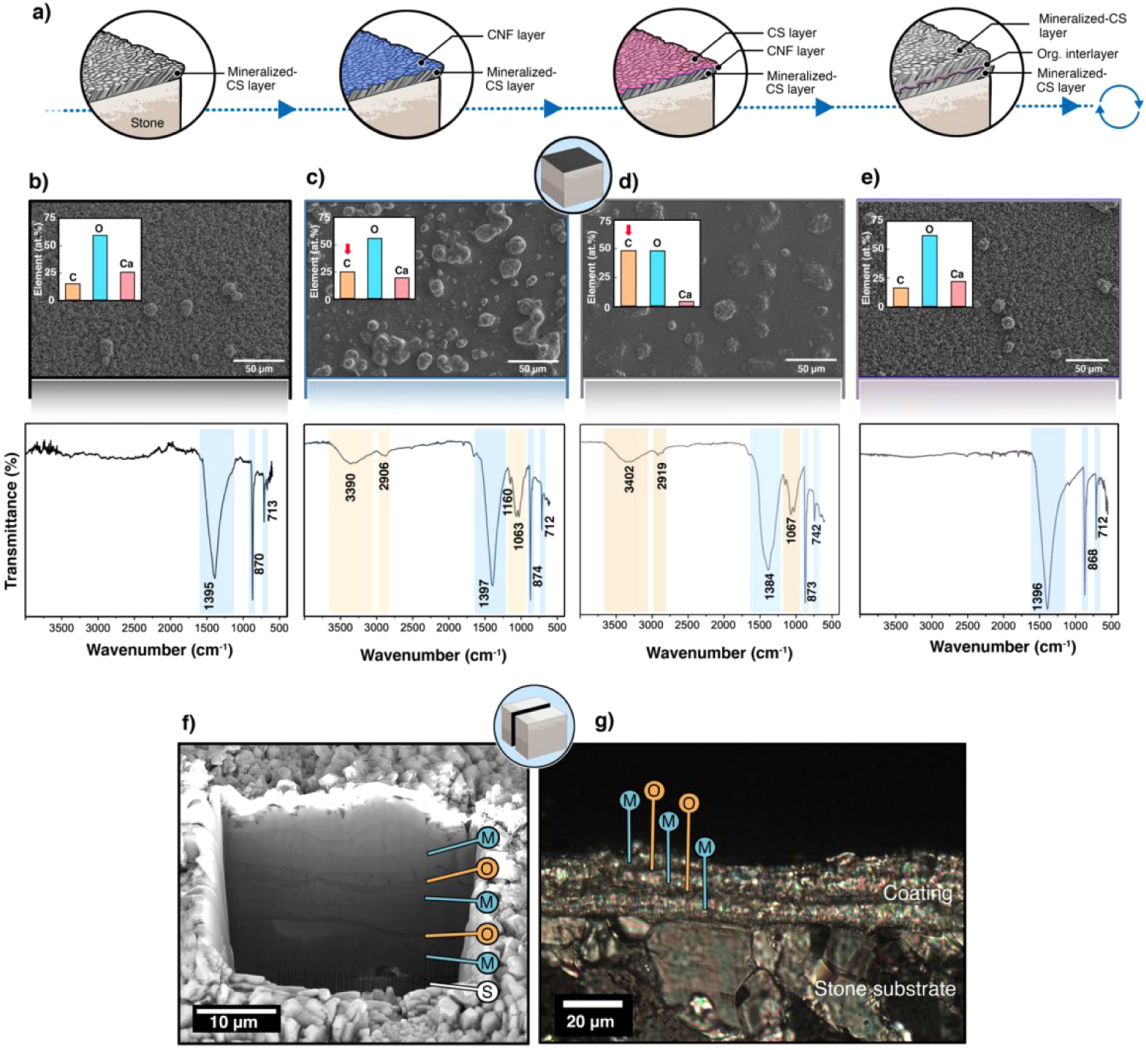
Layer-by-layer fabrication and characterization of mineralized composite multilayer coatings. (a) Schematic representation of the sequential deposition process used to build the multilayered structure consisting of alternating mineralized chitosan (CS) and cellulose nanofibril (CNF) layers on the stone substrate. (b–e) Scanning electron microscopy (SEM) images showing the surface morphology of each layer: (b) mineralized-CS, (c) CNF layer, (d) CS layer, and (e) final mineralized-CS layer. The insets in (b–e) show the elemental semiquantification obtained via energy-dispersive X-ray spectroscopy (EDS), highlighting the relative concentrations of carbon (C), oxygen (O), and calcium (Ca) for each layer. FTIR spectra (bottom panels) confirm the presence of functional groups, including carbonate (CO_3_^2–^) and amide bands, and showing the sequential deposition of organic and mineral components. (f) Focused ion beam (FIB) cross-sectional SEM image showing stratification of the multilayered structure, with mineralized layers (M), organic layers (O, CNF/CS), and substrate (S). (g) Polarized optical microscopy (POM) image of a cross-section displaying birefringence patterns indicative of crystallographic orientation and confirming the organized, layered deposition of the mineralized composite on the stone substrate.

The FIB cross-sectional SEM image (Figure 2f) provided a detailed view of the CML architecture, revealing stratification of the mineralized layers (denoted as M) and chitosan–CNF layers (denoted as O). The well-defined interfaces between these layers underscored the successful sequential deposition of each component. Additionally, the POM image (Figure 2g) highlighted distinct birefringence patterns across the structure, indicating variations in the crystallographic orientation of the calcium carbonate crystals. These birefringence patterns, characterized by alternating bright and dark regions, are indicative of anisotropic crystalline domains where the alignment of the crystals varies across the layers. Such patterns suggest a highly ordered layer-by-layer arrangement within mineralized CS layers. The observed birefringence confirmed the crystalline nature of the CaCO_3_, while the clear boundaries between the stone substrate, chitosan–CNF layers, and mineralized overlayers further confirmed the coherent deposition process, resulting in a composite multilayered system.

Although dedicated scalability tests were not performed, the decoupled polymer delivery-CO_2_ carbonation process exhibits characteristics that are compatible with extension to larger areas and nonplanar substrates. In particular, mineralization is driven by CO_2_ diffusion in the gas phase rather than by a directional line-of-sight deposition process, which reduces the sensitivity to substrate orientation and geometry. Qualitative support for this applicability is provided by mineralization experiments performed on a small nonplanar marble object, which resulted in predominantly continuous CaCO_3_ coverage over the exposed surface (Figure S7, Supporting Information). Systematic scalability and thickness-uniformity studies will be addressed in future work.

### 3.3 Mechanical Properties of the Composite Multilayer Coatings (CMLs)

Samples were prepared both perpendicular and parallel to the layered structure for mechanical characterization, allowing for the measurement of mechanical properties both in the cross-section and along the surface. The number of samples used in each mechanical test was optimized to ensure representativeness while minimizing redundant measurements. Cross-sectional analyses focused on X2 CML and X3 CML as representative configurations of the multilayer buildup, as this geometry probes the integrated mechanical response of the full stack, and higher layer numbers did not yield additional resolvable differences within the experimental uncertainty. In contrast, surface analyses included all variants (X2–X5 CML) to capture the progressive evolution of the mechanical properties with increasing number of layers.

*i) Cross-sectional:* The mechanical properties of the CMLs were assessed along the surface normal direction, with the indenter probing across the stacked architecture rather than along it, using nanoindentation and Peak Force Quantitative Nanomechanics (PF-QNM), as shown in Figure 3. The X2 CML and X3 CML samples, based on two and three mineralization cycles, respectively, comprise alternating organic-rich and mineralized CaCO_3_ layers that mimic the layered structural motif of biogenic composites. To contextualize their mechanical response, the hardness (*H*) values were compared with those of *Haliotis ovina* nacre, which serves as a well-established mechanical reference for hierarchical layered composites. This comparison allows the mechanical behavior of the CMLs to be contextualized in terms of architecture-driven toughening rather than compositional identity. In the transverse section (Figure 3a), the X2 CML and X3 CML samples exhibited hardness values of 2.8 ± 0.2 GPa and 2.8 ± 0.1 GPa, respectively, closely matching *Haliotis ovina* nacre (3.1 ± 0.1 GPa). These hardness values are also comparable to those of prismatic-types biogenic minerals found in mollusk shells, which typically exhibit hardness values ranging from 1 to 4 GPa.^44,57–59^ Cross-sectional SEM micrographs (Figure 3b) confirm that both coatings exhibit localized plastic deformation around indentations, with minimal radial cracking. This response is comparable to nacre, where the intercalated organic–inorganic layers act as energy dissipators, enhancing fracture resistance.^28,39^

**Figure 3:**
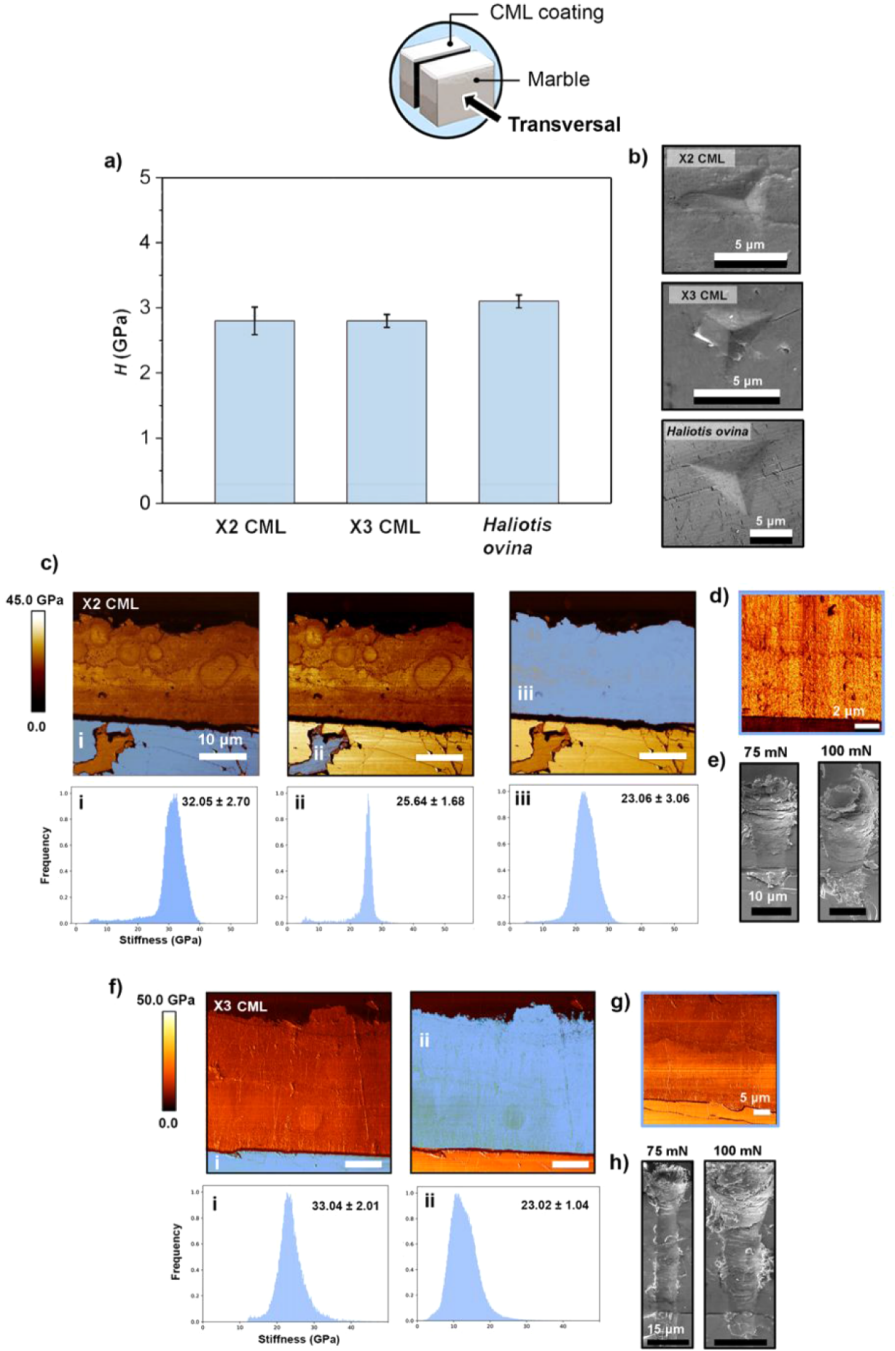
Cross-sectional (i.e., transverse) mechanical properties and structural integrity of the composite multilayer coatings (CMLs). (a) Nanoindentation hardness (*H*) measured in the transverse cross-section of X2 CML, X3 CML, and the biogenic reference *Haliotis ovina* nacre. Error bars represent standard deviations from at least ten independent measurements. (b) SEM images of representative nanoindentation imprints in the transverse cross sections of X2 CML, X3 CML, and *Haliotis ovina* nacre, showing comparable deformation behavior and the absence of extensive cracking. (c) Peak Force Quantitative Nanomechanics (PF-QNM) stiffness maps of the transverse cross-section of X2 CML, showing (i) the marble substrate, (ii) the marble/coating interface, and (iii) the mineralized CaCO_3_ layer; corresponding stiffness histograms are shown below each map. (d) Higher-magnification PF-QNM stiffness map of X2 CML, emphasizing local stiffness variations within the multilayer architecture. (e) SEM images of nanoscratch tracks across the substrate/CML interface of X2 CML acquired at applied normal loads of 75 mN and 100 mN, demonstrating coating cohesion and resistance to delamination. (f) PF-QNM stiffness maps and corresponding stiffness histograms of the transverse cross-section of X3 CML, showing (i) the marble substrate and (ii) the mineralized coating layer. (g) Higher-magnification PF-QNM stiffness map of X3 CML, illustrating the spatial homogeneity of the mineralized layer; (h) SEM images of nanoscratch tracks across the substrate/CML interface of X3 CML recorded at applied loads of 75 mN and 100 mN, highlighting the mechanical integrity of the multilayer coating under increasing loads.

PF-QNM stiffness mapping (Figure 3c and d) provided spatially resolved insights into the hierarchical mechanical properties of the CML coatings and their interaction with the marble substrate. The stiffness distribution within the calcite-based layers of X2 CML (∼23.1 ± 3.1 GPa) (Figure 3ciii) and X3 CML (∼23.0 ± 1.0 GPa) (Figure 3fii) closely resembles that of the calcitic grains in the marble substrate (∼25.6 ± 1.7 GPa) (Figure 3cii). This mechanical compatibility promotes a coherent interface between the protective coating and stone, which is essential for long-term durability and stress transfer efficiency. The underlying marble substrate also contains dolomite regions with higher stiffness values (29.4–35.1 GPa) (Figure 3c, *Panel i*). At higher magnifications, Peak Force mapping (Figure 3d), reveals localized stiffness variations within the CML, attributable to the alternating organic-rich and inorganic layers. This hierarchical architecture, characterized by the intercalation of flexible and stiff layers, is essential for dissipating mechanical stress and enhancing the toughness of the system. It is worth noting that the mechanical properties discussed here were derived from the transverse section analysis. In this orientation, the indenter probes across the stacked multilayer architecture rather than along it, indicating that the response reflects the integrated behavior of the multilayer as a composite rather than a layer-by-layer contribution. This may explain the similar mechanical values observed for X2 and X3 CMLs, as the cross-sectional geometry can mask subtle differences in multilayer composition or thickness by averaging their individual mechanical responses.

The nanoscratch tests performed with constants loads of 75 mN and 100 mN across the substrate/CML interfaces provided insights into the mechanical response and failure mechanisms of the X2 CML and X3 CML (Figure 3e and h, respectively). Both coatings exhibited shallow scratch tracks with no interlayer failure, indicating strong adhesion and effective stress distribution. Under higher loads (100 mN), localized material removal was observed at the interfaces; however, no catastrophic delamination occurred. This suggests that the cohesive strength of the multilayers is strong enough to inhibit crack propagation. The absence of delamination or cohesive failure suggests that the interlayer within the CML coatings effectively dissipates mechanical stresses, thereby preventing crack propagation and maintaining the structural integrity of the coating.

*ii) Surface analysis:* the hardness values measured in this orientation were slightly lower than those observed in the cross-sectional evaluation, with no significant differences as the number of layers increased (Figure 4a). This difference may be attributed to variations in the mechanical response of the coating when probed in cross-sectional and surface orientations. In cross-sectional evaluations (Figure 3), the indenter interacts more directly with the multilayer structure, as the layers are aligned perpendicularly to the indentation axis, potentially enhancing the resistance to localized deformation. Additionally, the interfaces between layers could act as barriers to plastic deformation, while residual stresses within the coating may also contribute to increased hardness. In contrast, the surface measurements primarily capture the response of the uppermost layers. The X2 CML and X3 CML samples exhibited hardness values of 2.2 ± 0.2 GPa and 2.1 ± 0.3 GPa, respectively, while the X4 CML and X5 CML samples showed values of 1.9 ± 0.2 GPa and 2.1 ± 0.1 GPa. Compared to the surface of the *Haliotis ovina* nacre (2.7 ± 0.2 GPa), these values are slightly lower but still fall within the typical values observed for prismatic biogenic minerals found in mollusk shells.^60,61^ It is important to highlight that these results pertain to the properties of a coating rather than a bulk material. While achieving high hardness, flexibility, and resistance to cracking is a general challenge for all materials, these factors are particularly critical in multilayered coatings, where each layer has a reduced thickness (typically 4–6 μm) and distinct structural constraints.

**Figure 4:**
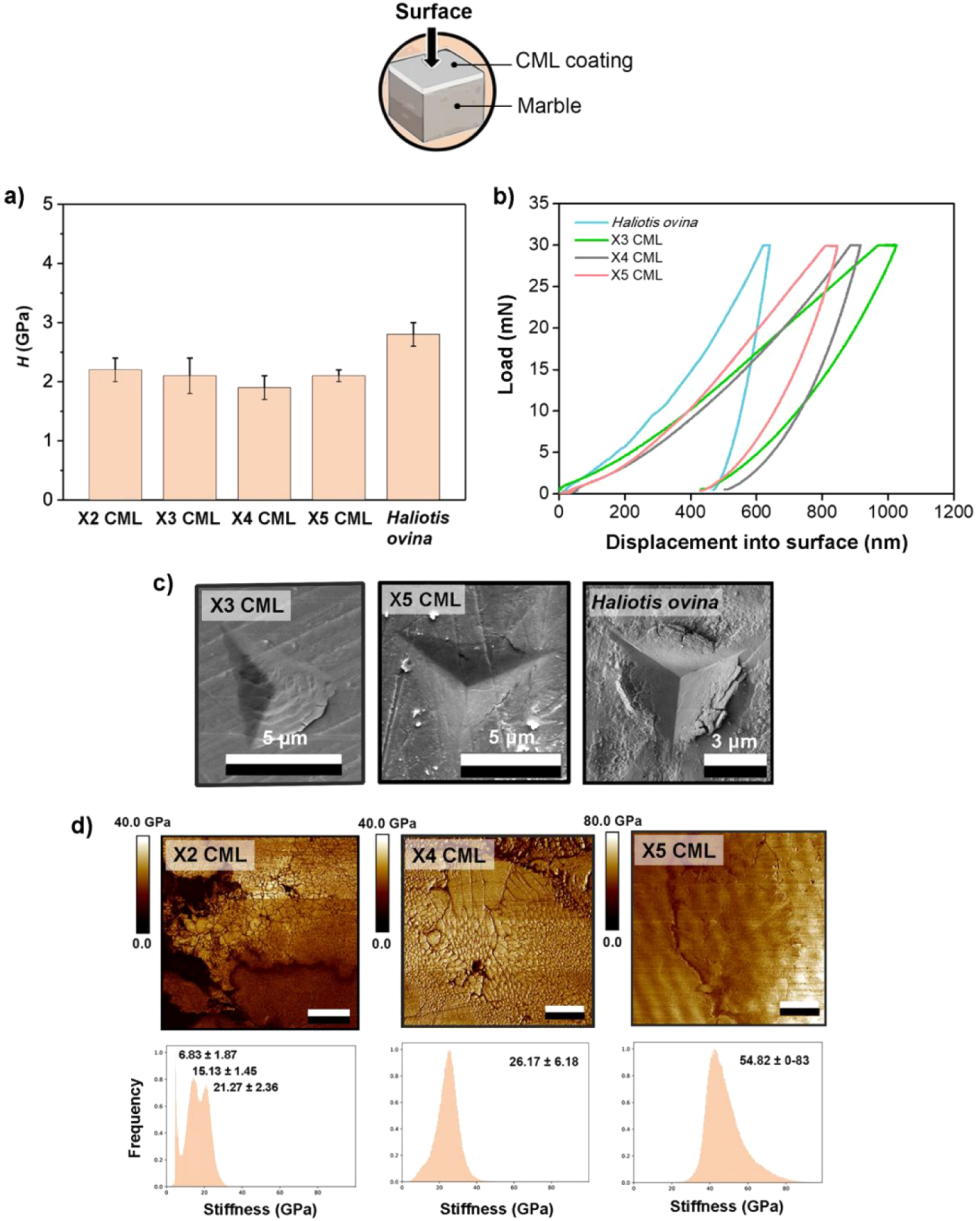
Nanoindentation hardness values (*H*) of the composite mineralized layers (CMLs) measured at the surface for X2, X3, X4, and X5 CMLs, compared to the reference value for *Haliotis ovina* nacre. CMLs refer to composite mineralized layers, and the number (X2, X3, etc.) indicates the number of mineralization cycles used to create the structure. (b) Load–displacement curves showing the mechanical response of the X3, X4, and X5 CMLs alongside *Haliotis ovina* nacre. (c) SEM images of the indentation imprints of the X3, and X5 CMLs as well as *Haliotis ovina* nacre, highlighting the morphological differences. Scale bars: 5 μm for CMLs, 3 μm for *Haliotis ovina*. (d) Peak force quantitative nanomechanical mapping (PF-QNM) images of stiffness for X2, X4, and X5 CMLs (Scale bar= 8 μm), showing stiffness distributions and their corresponding histograms.

While hardness values remained relatively constant across the surface of the CML samples, the reduced modulus (*E^r^*), as obtained by nanoindentation, increasing significantly with the number of layers. The X3 CML sample reached 12.2 ± 1.6 GPa, rising to 32.1 ± 3.6 GPa for the X4 CML sample, and further to 41.0 ± 2.4 GPa for the X5 CML sample (Figure 4b). These values are lower than the bulk elastic modulus of bulk *Haliotis ovina* nacre (68.9 ± 7.2 GPa), yet they remain within the typical range (10–40 GPa) reported for biogenic prismatic structures in mollusk shells and fall within the range of materials exhibiting nacre-like structures.^40,44^ This range is indicative of materials that balance stiffness and toughness, enabling resistance to mechanical damage while retaining a degree of flexibility.^54,60^ This upward trend indicates that the number of layers enhanced the stiffness (indentation modulus) of the coatings. These improved properties likely result from the hierarchical design shared by the CML coatings and other nacre-mimetic approaches.^38,39,61^ Thus, as in natural nacre where nanograins are bound by organic components such as proteins, which contribute to its mechanical robustness,^28,30,38^ the alternating organic and inorganic layers in the system, combined with the inclusion of PAA, may play a similar role. PAA likely interacts strongly with the CaCO_3_ layers, enhancing interlayer cohesion and acting as a binding agent, as observed in synthetic nacre systems.^39,61^ Moreover, the inclusion of chitosan and nanofibrils may aid in stress dissipation and crack deflection, as evidenced by the absence of significant crack propagation in the SEM images of the indentation imprints (Figure 4c). The limited crack formation and localized deformation suggest efficient energy dissipation, with the softer organic material facilitating crack deflection and strain absorption, thereby enhancing the resistance of the coating to mechanical damage.

In addition to the hardness and modulus trends, surface PF-QNM maps (Figure 4d) provide further insight into the nanomechanical heterogeneity of the multilayered coatings, revealing distinct stiffness distributions that reflect the hierarchical organization of the systems. The stiffness histograms for X2 CML exhibit a bimodal distribution, with lower stiffness values corresponding to the organic-rich components and higher values to the mineralized domains, emphasizing the presence of mechanically contrasting phases. This mechanical heterogeneity is consistent with the alternating soft and stiff layers characteristics of biomimetic nacre-like structures, where organic components serve as flexible interfaces facilitating stress dissipation. As the number of mineralization cycles increases (X4 CML and X5 CML), the distinction between these peaks diminishes, resulting in a more homogeneous stiffness distribution, reaching values of 54.82 ± 0.83 GPa for the X5 CML coating. This apparent shift could arise from an increased continuity and densification of the mineralized layers, leading to a progressive reinforcement of the system. However, it is also crucial to consider the influence of sample preparation and measurement conditions. Given that PF-QNM primarily probes the surface mechanical response with a typical penetration depth in the range of a few to several tens of nanometers, the measurements are highly surface sensitive. This is substantially shallower than the individual layer thicknesses in the CML system (approximately 4–6 μm), indicating that PF-QNM predominantly captures the mechanical properties of the outermost region. Depending on surface topography, this may include both organic and inorganic contributions, or in some cases, be confined to the mineral-rich surface. Therefore, the observed stiffness homogenization likely reflects intrinsic structural rearrangements at the top interface, although differences in surface exposure and local material composition may also contribute. Despite these potential methodological influences, the PF-QNM results align with the increasing indentation modulus observed in Figure 4b, reinforcing the hypothesis that the hierarchical organization of multilayered coatings contributes to their mechanical performance. Similar to natural nacre, where the organic phase acts as a mechanical modulator by enhancing toughness and crack resistance, the organic layers within the CML coatings likely play a key role in strain accommodation and energy dissipation. As mineralization progresses, these layers may become increasingly confined between stiffer domains, reducing their direct contribution to the measured stiffness while continuing to function as toughening agents at the interface. This mechanical confinement may limit their deformation under load but still allows them to dissipate energy and accommodate strain during the crack propagation.

### 3.4 Mechanical Durability and Surface Evolution of Composite Multilayer Coatings Exposed to Acidic Media

The mechanical durability of the CML coatings under acidic conditions was assessed by analyzing their mechanical properties and surface morphology before and after exposure to an acid environment. Specifically, the X4 CML sample underwent an accelerated aging test in a 24-h exposure to nitric acid (pH 5) to simulate the effects of acid rain and urban pollution on historical stone substrates.^62^ The X4 CML configuration was selected as a representative and conservative model system for acid durability testing, as it corresponds to an intermediate stage in the multilayer buildup where the key structural features governing chemical stability are fully developed. At this stage, the coating exhibited a continuous mineralized outer layer, multiple organic–inorganic interfaces, and sufficient overall cohesion to withstand chemical attack. Lower multilayer numbers (X2–X3) primarily differ in total thickness and reinforcement level, whereas higher multilayer numbers (X5) mainly increase the repetition of analogous structural motifs rather than introducing additional degradation mechanisms. Accordingly, testing X4 CML enables the assessment of the dominant acid-induced degradation pathways without the redundancy associated with evaluating multiple architectures expected to respond similarly. The impact of acid attack on the mechanical properties and surface morphology was analyzed through nanoindentation, SEM, and surface roughness mapping (Figure 5a–c).

**Figure 5:**
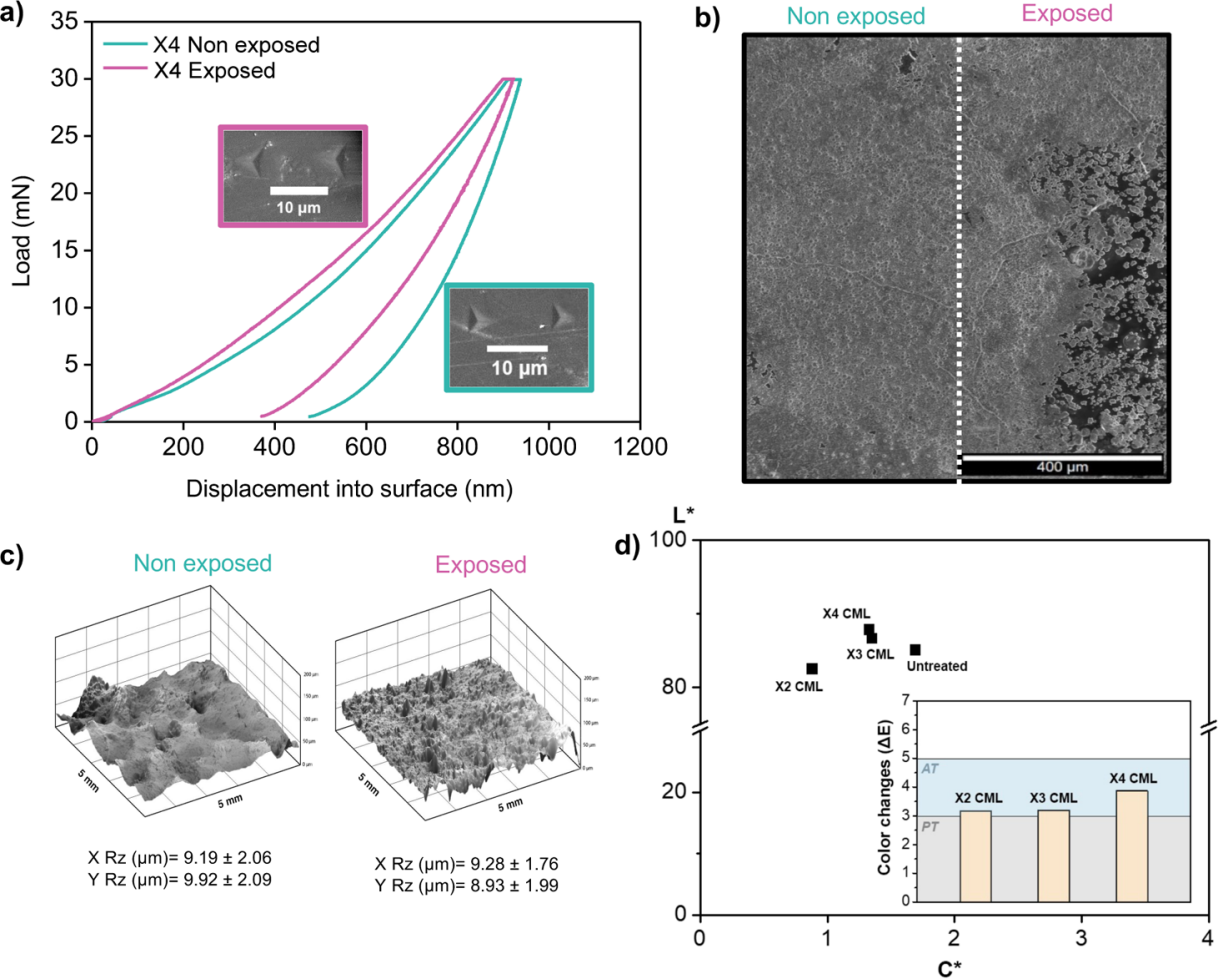
Comparative evaluation of X4 CML coatings before and after exposure to acidic conditions. (a) Nanoindentation load–displacement curves and corresponding indentation imprints (inset). (b) SEM micrographs of the coated surface compared with the nonexposed and acid-exposed areas. (c) 3D roughness analysis (Rz) in the *X*- and *Y*-directions of nonexposed and exposed samples. (d) Colorimetric parameters (lightness L* and chroma C*) for untreated marble and CML samples (X2, X3, X4), along with the total color difference (ΔE*ab), confirming the visual compatibility of the coatings after acid exposure.

Prior to acid exposure, the X4 CML displayed a well-balanced combination of hardness and elastic modulus, attributable to its hierarchical organic–inorganic structure. This multilayered design enabled effective stress dissipation, which enhanced the resistance of the coating to localized mechanical deformation. Following acid exposure, the coating maintained its structural coherence with only moderate changes in the mechanical behavior (Figure 5a). Thus, hardness values remained stable following acid exposure, indicating the retention of surface mechanical resistance. In contrast, the elastic modulus decreased from 41.0 to 32.1 GPa (≈21.7%), evidencing a partial loss of stiffness likely associated with acid-induced degradation of the inorganic phase (Figure 5a). This combination highlights the ability of the nacre-inspired architecture to balance mechanical reinforcement, chemical durability, and aesthetic compatibility. Notably, the indentation imprints showed no significant cracking or delamination, confirming that the multilayer architecture retained its mechanical integrity (Figure 5a, *inset*). This resilience further emphasizes the role of the hierarchical assembly in mitigating chemical degradation, highlighting the potential of bioinspired coatings to be used for heritage conservation.

The surface morphology underwent certain changes following acid exposure, as revealed by SEM analysis, which localized dissolution processes affecting the coating structure (Figure 5b). The initially fine-textured and relatively homogeneous surface of the pristine X4 CML, characterized by a granular mineralized matrix, exhibited local morphological changes after acid exposure. Although the overall peak-to-valley roughness (Rz) values remained statistically comparable (Figure 5c), 3D surface mapping revealed a shift in the surface texture with slightly more fragmented features and localized sharpness. This suggests that acid exposure induces subtle topographical changes that are likely associated with localized mineral leaching in the less compact inorganic regions of the coating. Despite these modifications, the CML coating preserved its cohesion and showed no evidence of detachment or collapse. These observations reinforce the effectiveness of the organic–inorganic interphase in maintaining the mechanical stability under chemically aggressive conditions.

It is worth noting that the mineralogical composition of the marble substrate plays a critical role in the chemical stability and interfacial behavior of protective coatings, particularly under acidic exposure. Dolomite (CaMg­(CO_3_)_2_) exhibits greater resistance to acid-induced dissolution than calcite (CaCO_3_),^63^ due to its lower solubility and the stabilizing effect of magnesium within the crystal lattice. In contrast, calcitic substrates are more susceptible to proton-mediated dissolution when exposed to acidic agents such as nitric acid, which can accelerate surface degradation and potentially compromise coating adhesion and long-term mechanical integrity. Therefore, substrate mineralogy must be carefully considered when designing protective strategies for stone heritage, as variations in carbonate composition may critically influence coating performance under environmental stressors.

Potential color alterations following treatment can limit the applicability of coatings in situ, particularly in cultural heritage contexts where preservation of the original appearance is essential. Therefore, the color changes induced by the CML coatings were evaluated systematically. Colorimetric analysis showed that the X4 CML coated surface exhibited a lightness (L*) of 87.84 and a chroma (C*) of 1.33, compared to 85.09 and 1.69, respectively, for the native marble (Figure 5d). These values indicate a slight increase in luminosity and a modest reduction in color saturation. The total color difference (ΔEab = 3.87), however, remained well below the commonly accepted threshold for conservation treatments (ΔEab ≤ 5),^4^ confirming that the intervention did not results in perceptually disturbing changes. Additional measurements on samples with a lower multilayer buildup exhibited similarly low ΔEab values (ΔEab = 3.16, X2 CML, and ΔEab = 3.19 for X3 CML), further supporting the aesthetic compatibility of the coating system (Figure 5d). Moreover, coated surfaces exposed to chemically aggressive conditions showed no significant additional color change, indicating that the CML coating not only preserves the initial appearance upon application but also provides effective protection against acid-induced discoloration. Within the broader landscape of protective treatments for carbonate stones, the performance of CML coatings can be viewed as complementary to that reported for established approaches. Silane- and organosilicon-based systems are widely recognized for their effectiveness in preserving surface appearance,^11^ while mineral-based treatments have demonstrated strong chemical affinity and resistance in carbonate environments.^24^ In this context, the nacre-inspired multilayer architecture presented here combines moderate stiffness retention under acidic exposure with minimal chromatic alteration (ΔE*ab < 5), positioning it as a balanced strategy that integrates mechanical response, aesthetic compatibility, and chemical stability into a single coating design.

## 4 Conclusion and Outlook

In this study, we present a bioinspired strategy to fabricate hierarchical composite multilayer coatings (CMLs) designed for the protection of built cultural heritage. Inspired by natural biominerals such as nacre, CMLs were constructed through the alternating deposition of mineralized CaCO_3_ and organic layers composed of chitosan and cellulose nanofibrils (CNFs) guided by poly­(acrylic acid) (PAA) as a mineralization-directing agent over marble substrates. This layer-by-layer assembly yielded stratified coherent structures with robust interfacial adhesion to the stone substrate. Mechanical characterization revealed hardness and stiffness values comparable to those of natural composites, along with a progressive increase in the indentation modulus with a multilayer buildup. The coatings also demonstrated resistance to cracking and delamination under mechanical stress. Furthermore, chemical durability testing under mildly acidic conditions confirmed that the hierarchical structure maintains both mechanical integrity and visual compatibility with the substrate, two essential criteria for application in heritage conservation.

Building upon these findings, future work will focus on the systematic investigation of multilayer architecture parameters, including the number of layers, thickness ratios, and spatial distribution of organic and mineral phases, to further tailor the mechanical and chemical performance. Increasing the multilayer buildup may enable greater reinforcement, whereas modulating the composition and density of organic interfaces could enhance flexibility and crack resistance. Additionally, exploring alternative biopolymers or mineral phases may expand the functional versatility of coatings under diverse environmental and substrate conditions.

From an application perspective, the multilayer strategy introduced herein is compatible with scalable fabrication techniques and can be adapted to larger surface areas or more complex geometries. This can be achieved through the integration of deposition methods such as spray coating, printing-based technologies, or environmentally controlled processes (e.g., CO_2_ diffusion chambers). These approaches offer a pathway for the practical implementation of conformal high-performance coatings for architectural conservation. Overall, this study lays the foundation for the development of an emerging class of protective systems that unite structural precision, material functionality, and suitability for cultural heritage preservation.

## Supplementary Material



## Notes

Supplementary SEM images illustrating calcium carbonate mineralization under different polymer conditions, 3D surface profilometry data, optical and electron microscopy characterization of the marble substrate (POM, BSE–EDS, XRD), Raman spectroscopy confirming CaCO_3_ formation, and additional SEM and optical images demonstrating mineralization on nonplanar marble geometries (PDF
