# Supplementary material for: Nacre-Inspired Composite Coatings with Hierarchical Architecture for Durable Surface Protection
Source: Chem Mater. 2026 Feb 13;38(4):1821–35. doi: 10.1021/acs.chemmater.5c02825 (PMC12937189; doi:10.1021/acs.chemmater.5c02825)
Supplement: Supplementary file 1 [file cm5c02825_si_001.pdf]

Supporting Information

Nacre-inspired composite coatings with hierarchical architecture for durable surface protection

Aranzazu Sierra-Fernández[1,2,3], Diego Cortes[3], Miguel A. Monclus[4], Kenneth J.T. Livi[5,6], Michael Kappl[3], Stefan A.L. Weber[3,7,8], D. Howard Fairbrother[2*], Rafael Fort[1].*

[1] Institute of Geosciences (CSIC, UCM), C/ Severo Ochoa 7, Madrid 28040, Spain

[2] Department of Chemistry, Johns Hopkins University, 3400 N Charles Street, Baltimore, Maryland 21218, United States

[3] Max Planck Institute for Polymer Research, Ackermannweg 10, 55128 Mainz, Germany

[4] IMDEA Materials Institute, C/Eric Kandel 2, Getafe, 28906 Madrid, Spain

[5] Department of Earth and Planetary Sciences, Johns Hopkins University, Baltimore, Maryland 21218, United States

[6] Materials Characterization and Processing, 800 Wyman Park Dr., Johns Hopkins University, Baltimore, Maryland 21211, United States

[7] Department of Physics, University of Mainz, Staudingerweg 7, 55128 Mainz, Germany

[8] Institute for Photovoltaics, University of Stuttgart, Pfaffenwaldring 47, 70569 Stuttgart, Germany

* Corresponding authors: a.sierra@nanogune.eu; howardf@jhu.edu

1. Supplementary figures

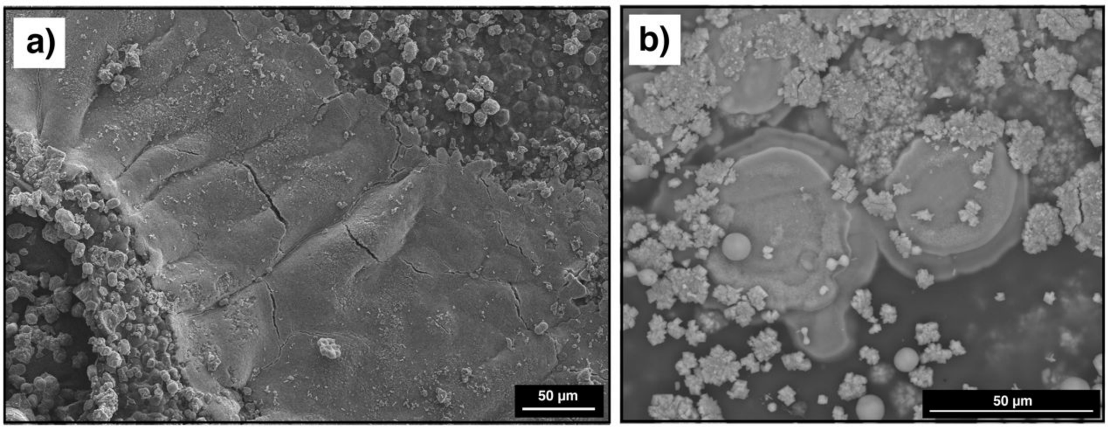

Figure S1. (a) Large scale SEM image showing a non-uniform chitosan layer leading to disorganized calcium carbonate deposition. (b) Detailed SEM image showing irregular crystal formation on an unevenly chitosan coated marble surface.

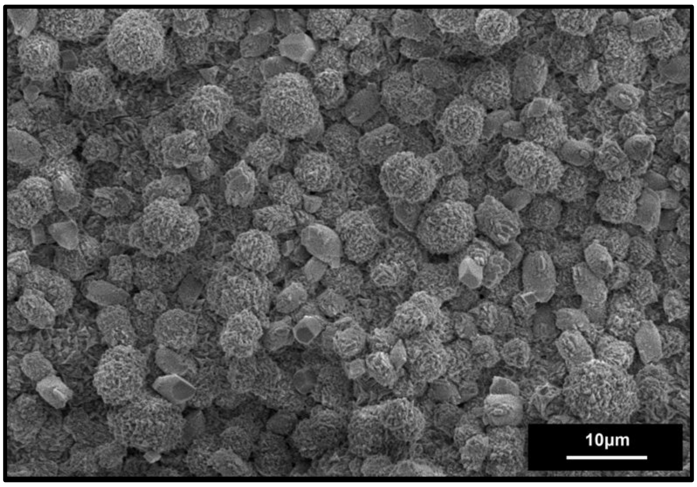

Figure S2. SEM image of calcium carbonate mineralization in the presence of 1 g/L poly-L-glutamic acid (PGlu).

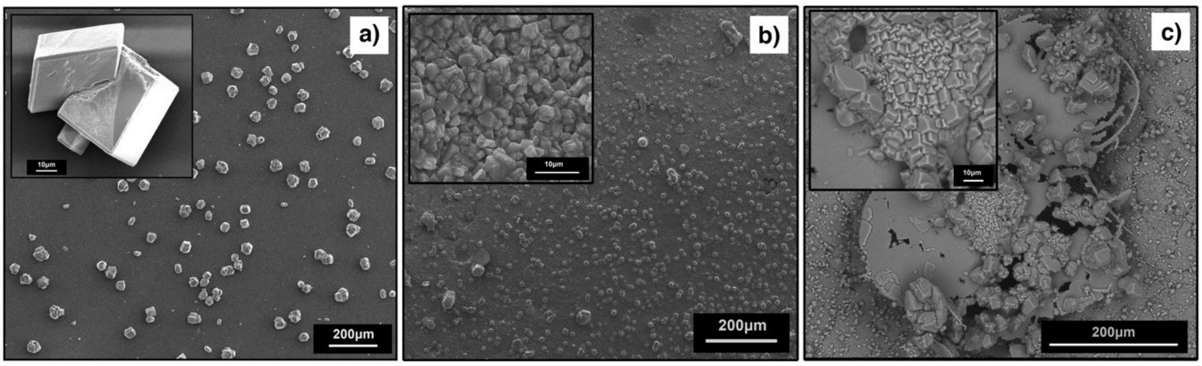

Figure S3. *SEM images showing the effect of poly(acrylic acid) (PAA) concentration on calcium carbonate mineralization.* (a) Mineralization without PAA, resulting in well-defined, isolated calcium carbonate crystals. (b) Mineralization in the presence of 40 µg/mL PAA, showing more evenly distributed finer crystals across the surface. (c) Mineralization at a high PAA concentration (200 µg/mL), resulting in larger, more angular calcium carbonate crystals with a blocky morphology. The insets in each panel provide higher-magnification views of the crystal structures. Scale bars= 10µm.

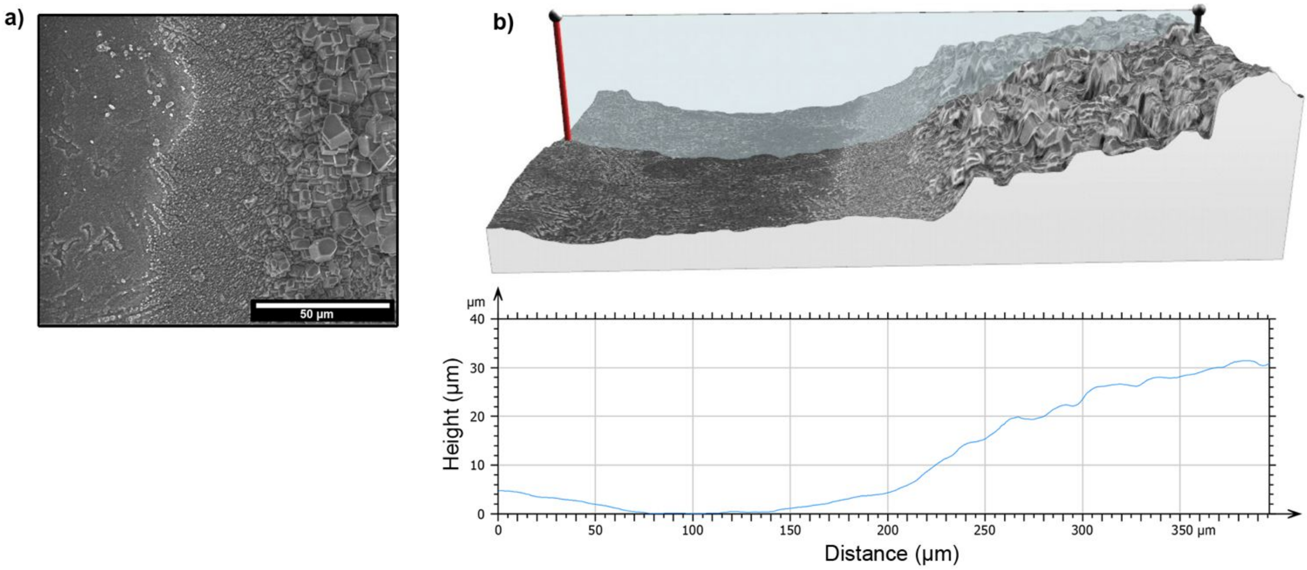

Figure S4. *SEM image and 3D surface profile of mineralized CS-$CaCO_3$ on a calcite substrate* obtained using a mineralization solution containing calcium chloride (20 mM $CaCl_2$) and polyacrylic acid (40 µg/mL PAA). (a) SEM image showing the interface between mineralized and non-mineralized regions. The right side of the sample, exposed to the mineralization solution, exhibits crystal growth and increased surface roughness, in contrast to the smoother area on the left of the sample, which was not exposed. (b) 3D surface roughness profile across the interface, highlighting the progressive increase in surface height due to mineral deposition.

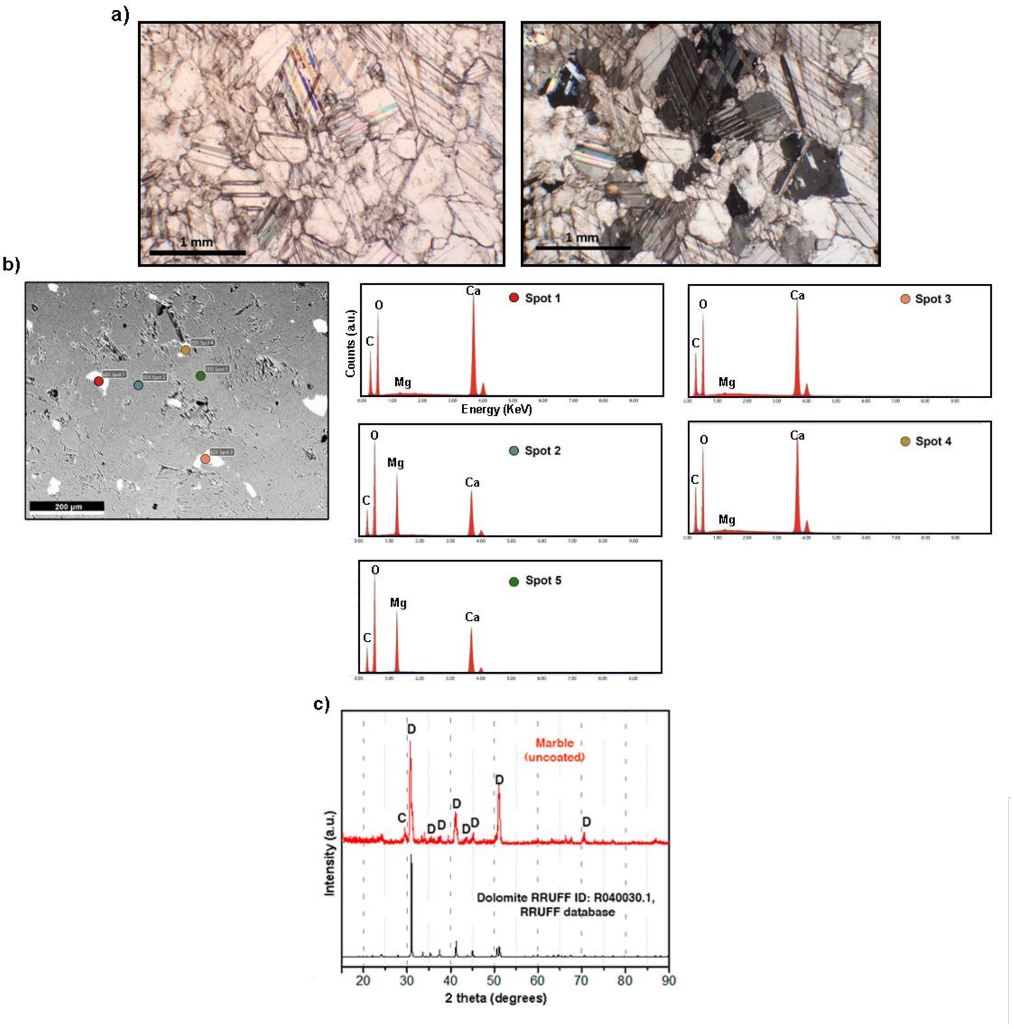

Figure S5. Characterization of the marble substrate with dolomitic composition. (a) Polarized optical microscopy (POM) images of thin sections from the stone sample, showing the crystalline texture and birefringence typical of carbonate minerals. The image on the left was taken under plane-polarized light whereas the right image was taken under crossed-polarized light (crossed nicols); (b) Backscattered electron image (BSE) of the same sample with corresponding energy-dispersive X-ray spectroscopy (EDS) point analyses (Spots 1-5), showing the coexistence of calcitic and dolomitic regions. Spectra shows areas rich in calcium (Ca), consistent with calcite ($CaCO_3$), and others with significant magnesium (Mg) content alongside calcium, corresponding to dolomite ($CaMg(CO_3)_2$); (c) X-ray diffraction (XRD) pattern confirming the presence of dolomite through characteristic peaks of $CaMg(CO_3)_2$. C (calcite), D (dolomite).

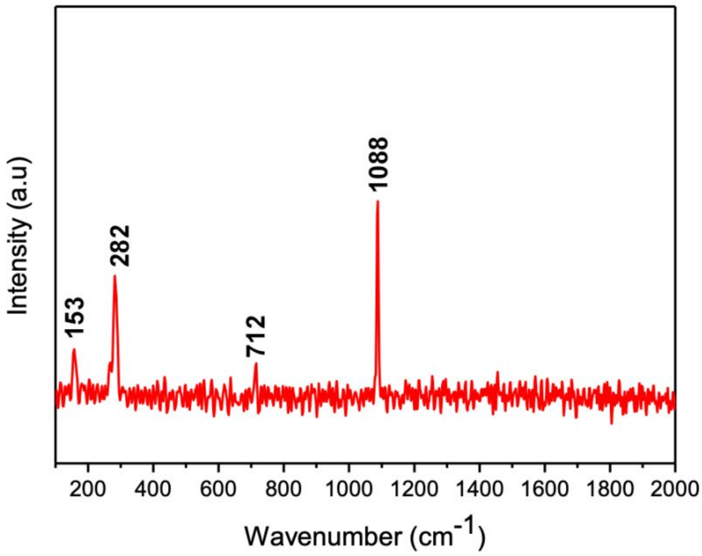

Figure S6. *Raman spectrum of calcium carbonate mineralization on top of the marble substrate.* The spectrum shows characteristic peaks at 153 cm^{-1}, 282 cm^{-1}, 712 cm^{-1}, and 1088 cm^{-1}, confirming the presence of calcium carbonate (calcite) on the coated surface. The sharp peak at 1088 cm^{-1} corresponds to the symmetric stretch (ν_1) mode of the carbonate ion (CO_3^{2-}).

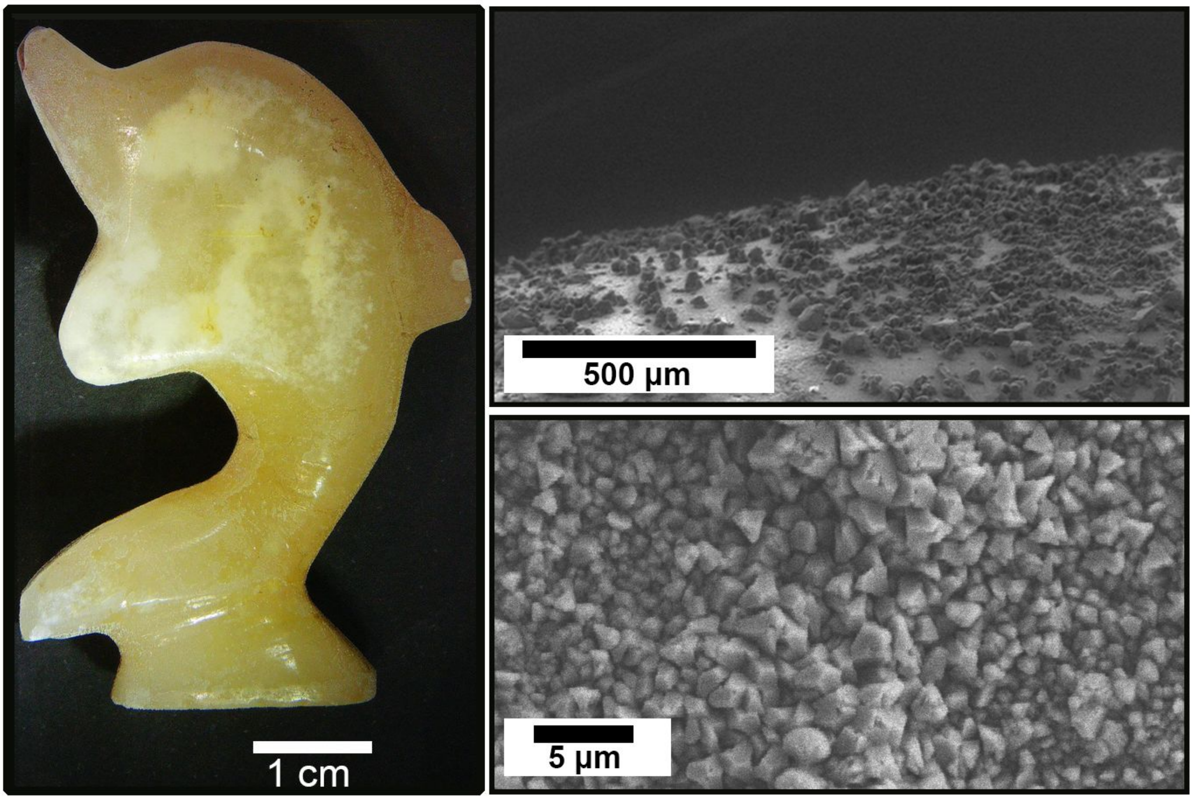

Figure S7. Optical image (left) and SEM micrographs (right) of a small marble object with a non-planar geometry after $CaCO_3$ mineralization using the standard PAA concentration (40 µg mL^{-1}). The optical image shows continuous surface coverage over the curved and inclined regions. Low-magnification SEM (500 µm) indicated mineral deposition without evident localized overgrowth, whereas higher-magnification SEM (5 µm) revealed a granular $CaCO_3$ morphology comparable to that observed on planar marble substrates. These observations provide qualitative support for the applicability of the CO_2 diffusion-driven mineralization process to substrates with complex geometries.
